# Measuring genetic diversity across populations

**DOI:** 10.1371/journal.pcbi.1012651

**Published:** 2024-12-04

**Authors:** Niloufar Abhari, Caroline Colijn, Arne Mooers, Paul Tupper

**Affiliations:** 1 Department of Mathematics, Simon Fraser University, Burnaby, BC, Canada; 2 Department of Biological Sciences, Simon Fraser University, Burnaby, BC, Canada; Max Planck Institute for Evolutionary Biology, GERMANY

## Abstract

Diversity plays an important role in various domains, including conservation, whether it describes diversity within a population or diversity over a set of species. While various strategies for measuring among-species diversity have emerged (e.g. Phylogenetic Diversity (PD), Split System Diversity (SSD) and entropy-based methods), extensions to populations are rare. An understudied problem is how to assess the diversity of a collection of populations where each has its own internal diversity. Relying solely on measures that treat each population as a monomorphic lineage (like a species) can be misleading. To address this problem, we present four population-level diversity assessment approaches: Pooling, Averaging, Pairwise Differencing, and Fixing. These approaches can be used to extend any diversity measure that is primarily defined for a group of individuals to a collection of populations. We then apply the approaches to two measures of diversity that have been used in conservation—Heterozygosity (Het) and Split System Diversity (SSD)—across a dataset comprising SNP data for 50 anadromous Atlantic salmon populations. We investigate agreement and disagreement between these measures of diversity when used to identify optimal sets of populations for conservation, on both the observed data, and randomized and simulated datasets. The similarity and differences of the maximum-diversity sets as well as the pairwise correlations among our proposed measures emphasize the need to clearly define what aspects of biodiversity we aim to both measure and optimize, to ensure meaningful and effective conservation decisions.

## Author summary

Measuring diversity has several implications including conservation prioritization. However, accurately assessing genetic diversity across multiple populations while considering both within and among population variations is often overlooked. Our study addresses this gap by introducing four novel methods, allowing a better understanding of measuring diversity across populations from different perspectives. We applied these methods to simulated data and data from Atlantic salmon populations. The results sometimes disagree on which populations are most important for conservation, highlighting the need for clear biodiversity goals in conservation decisions. It’s important to note that there isn’t a single correct answer to the question of how to measure diversity, and the best approach is likely to depend on the conservation priorities and criteria under consideration. By understanding the reason behind these differences, we can make more informed choices to protect our natural world. This research contributes to a broader understanding of diversity and can help guide areas where measuring diversity is important.

## Introduction

In today’s rapidly changing world, preserving biodiversity at multiple scales is a matter of utmost importance [1]. In particular, there have been renewed calls by researchers and international entities to protect intra-specific genetic diversity [2–7]. An analysis by Ceballos et al. reveals a severe and urgent mass extinction crisis, extending beyond species extinctions to widespread anthropogenic-driven population declines, with potentially profound consequences for ecosystem functioning and the services essential for humans [8, 9]. The assumption is that the loss of genetically distinct populations represents the loss of genetic information necessary for species to adapt and survive in changing environments [8]. While measuring genetic diversity of sets of populations has many applications, including understanding evolutionary processes [10, 11], disease resistance [12, 13], and ecosystem functioning [14], our focus here is on its key role in aiding conservation efforts amidst this pressing global challenge.

Researchers have come up with many different ways to measure diversity that may be applied at different levels (i.e. populations, species, higher taxa) [15–19]. One well-known task is to measure the diversity of a group of entities, for example, a group of species. Typically in such cases, each species is considered invariant, and so is represented by a single individual, a sequence, or a genome. One approach measures the *Phylogenetic Diversity* (PD) of a group of the species: a phylogenetic tree is constructed for all the species, and the diversity of a subset of the species is defined as the total length of the minimal subtree connecting them [15, 20]. Another method that is distinct but related is *Split System Diversity* (SSD), where the diversity of a subset of species is the fraction of measured traits (e.g. Single Nucleotide Polymorphisms (SNPs)) where all observed states (e.g. 0 and 1 in SNPs) are represented [21–23]. These two methods correspond closely in the case of the infinite sites model in population genetics. Split system diversity has the advantage that it can sensibly be applied when a phylogeny is not an appropriate model of the relationships among entities, e.g. if multiple populations within a species are being assessed. SSD can be used for any collection of traits, e.g. a vector of SNP sites, whether each collection represents a single species or each collection represents a single individual from a population (or individuals across multiple populations) [18, 22, 23].

We note that none of these approaches consider the population sizes of the entities being compared, nor do they consider any variation within them. This stands in contrast to studies like that of Luck et al. [24], which assesses the diversity of sets of populations using population size, number, distribution, and genetic composition of component populations. Another study by Hoban et al. [3] proposes some indicators of within-population diversity, including the effective population size. In this latter study, the authors suggest that for genetically distinct populations (which they do not clearly define), a population needs immediate conservation attention if its effective population size goes below 500.

A distinct perspective emphasizes species abundance as the primary datum of importance [25]. In the most basic forms, only the proportion of all individuals in each group matters, where a group could be genetically distinct individuals labelled as a single population. Entropy (and the exponential of entropy) is the main example here [26]; entropy is in the more general class of Hill numbers [27]. Entropy measures the diversity in a system, based on the proportions of individuals in different groups [26]. Hill numbers are a broader class of diversity indices that generalize entropy to account for different weights on rare versus common species or groups [27]. In this kind of diversity measure, the dissimilarity between species does not factor in except in the definitions of what the underlying groups are, though later work has found ways to include it naturally [25, 28–30]. Kosman [29] reviews the problem of making inferences about variation within and among populations, and explores measures of diversity that are based on the frequencies of genotypes in a population as well as some measures based on dissimilarity between operational units (e.g. individuals, or populations).

Although, in general, it has been difficult to develop a measure that includes both species abundance and phylogenetic history, Chao et al. [27] proposed a diversity metric that is sensitive to both. Their suggested metric calculates the “mean effective number of species” within a specified time interval on a tree. The multiplication of this mean by the duration of the interval provides a measure of the “branch diversity” exhibited by the phylogenetic tree throughout that time frame. This study extends the conventional phylogenetic methodology, originally centred on total phylogenetic length, by incorporating species abundances [27].

However, the diversity of a group of individuals, either individual organisms in a population or individual species in a clade (with or without factoring in abundance) is just one instance where we might want to assess diversity for e.g. conservation reasons. An understudied problem is how to assess the diversity of a collection of populations, each of which is composed of individuals. Several years ago, Petit et al. [31] introduced a method to measure the contribution of individual populations to overall genetic diversity. They used Nei’s genetic diversity [32] and compared diversity with and without each population. This method considered both population divergence and internal diversity. They proposed comparing each population’s contribution to the mean contribution of all populations to identify those warranting conservation attention due to their high genetic contributions. Later, Caballero and Toro [17] also considered the contribution of individual populations to measure the global diversity of a collection of populations. They highlighted that relying solely on individual-level diversity measures in conservation prioritization can lead to misleading results. They emphasized the importance of considering the global diversity of the collection of populations, which requires incorporating both within-population and between-population variability into conservation decisions.

Following this perspective, we present four approaches, *Pooling, Averaging, Pairwise Differencing*, and *Fixing*, for assessing the diversity of a set of populations. While we focus on two specific measures (heterozygosity and SSD, see below), our four approaches could be built upon any measure of diversity defined on a set of individuals. Pooling involves taking a measure that is originally defined for a single population and applying it to a combined or pooled set of populations. The averaging method applies the single-population measure to each of the populations in the set and computes the average of the obtained results. The pairwise differencing method applies the single-population measure to each of the populations and then measures the variability of the measures over all the populations. Lastly, the fixing method estimates the expected diversity after “fixation” (see below) has occurred in each of the populations at all sites.

These four approaches yield different results and may lead to different conservation decisions. To illustrate the problem, suppose we want to assess diversity with respect to one single locus with two alleles (denoted 0 and 1). Each population of such individuals can be characterized by the fraction of individuals that have the allele 1 (i.e. *p*). If we have two populations one of which has *p* = 0.1 and the other *p* = 0.5, and we have to prioritize just one for conservation, then an excellent choice for maximizing diversity is to select the population with *p* = 0.5.

On the other hand, suppose now we have to prioritize the conservation of two islands, A and B, each of which has two equally-sized populations of organisms. On the first island, the fractions of allele 1 in the two populations are *A* = (0.1, 0.9). In the second island, the fractions are *B* = (0.5, 0.5). Which island’s preservation should be prioritized in the interest of maintaining within-species genetic diversity? One answer to the question is that the two islands are equivalent: the total fraction of allele 1 is 0.5 on both islands. Another answer is to prioritize island A as the populations there are actually different from each other, whereas, on island B, they are all the same. From another perspective, we might prefer island B, if we thought that only one population on the island we saved was likely to survive into the future. There are some value judgments here on how to measure the biodiversity of a collection of populations, even when we agree on how to measure the biodiversity of a single population.

To see how these issues play out on a data set of conservation importance, we applied our four methods to Heterozygosity (Het), a widely used measure for evaluating genetic variation within a single population. Our proposed Het-based population diversity measures were applied on a dataset comprising SNPs across multiple loci from 50 distinct anadromous Atlantic salmon populations [33]. We compared our population diversity measures using differently-sized subsets of salmon populations, and computed maximum-diversity set(s) using the different diversity measures.

We also applied our proposed approaches to SSD, an alternative diversity metric closely related to heterozygosity. The same comparisons were conducted between all four SSD-based measures and between the SSD-based and Het-based measures. For both measures, we also consider randomized and simulated data to explore the generality of our observations.

## Methods

In what follows, we assume *X* is a finite set of *n* individuals (e.g. taxa/species), where each individual is represented by a vector of 0s and 1s. Each element of the vector corresponds to a distinct site, which refers to a specific location or position within a DNA sequence, representing two variations of an allele as either 0 or 1. For the clarity of the equations and our explanations, we often focus on a single site. Later in the paper, we compute the summation across all sites when reporting the correlation analysis results, using the *Pearson Correlation Coefficient (r)* (See the S1 Text, where we show that the correlation between measures at a single locus is preserved when we sum over loci.). Moreover, we assume *Y* is a finite set of *m* populations, where all populations have the same size. To maintain clarity, the notation 1_*E*_ will be used to indicate a value of 1 when condition *E* is true and 0 when *E* is false.

### Heterozygosity: From individuals to populations

Traditionally, expected heterozygosity is the probability that a diploid individual carries two distinct alleles at a particular genetic locus. We consider heterozygosity as the probability that two random individuals in a population have different alleles at a given site, assuming uniform draws (with replacement); this is motivated by the structure of the data that we use. We provide three different equivalent expressions for the heterozygosity of a single population, each of which is useful in different contexts. We then apply the four proposed methods to extend them to collections of populations, each of which captures diversity from a different perspective.

Let *X* = {*x*_*i*_|*x*_*i*_ ∈ {0, 1}, *i* = 1, …, *n*} denote whether individual *i* has the 0 or 1 allele at a particular locus, so that $p=\frac{1}{n}\sum_{i=1}^{n}x_{i}$ is the fraction of individuals with the 1 allele. Table 1 (Het_ind_ in the first row) shows three equivalent expressions for heterozygosity at this locus. The first is in terms of *p* and is the standard formulation for the probability of getting two different alleles 2*p*(1 − *p*). The second expression is obtained by observing that there are *n*^2^ ways of choosing *x*_*i*_ and *x*_*j*_, each with probability 1/*n*^2^, and they will be different if and only if (*x*_*i*_ − *x*_*j*_)^2^ = 1, and the same if and only if (*x*_*i*_ − *x*_*j*_)^2^ = 0. In other words, ${\left(x_{i}-x_{j}\right)}^{2}=1_{x_{i}\neq x_{j}}$. The third follows similarly: for each *i* and *j*, $1-x_{i}x_{j}-\left(1-x_{i}\right)\left(1-x_{j}\right)=1_{x_{i}\neq x_{j}}$, as can be checked by going through all the cases.

**Table 1:** Different representations of Het score and SSD score for a set of individuals.

	In terms of *p*	In terms of (*x*_*i*_ − *x*_*j*_)	Linear in *x*_*i*_
Het_ind_	2*p*(1 − *p*)	$\frac{1}{n^{2}}\Sigma_{i,j}{\left(x_{i}-x_{j}\right)}^{2}$	$\frac{1}{n^{2}}\Sigma_{\begin{array}{c} i,j\\ i\neq j \end{array}}[1-x_{i}x_{j}-\left(1-x_{i}\right)\left(1-x_{j}\right)]$
SSD_ind_	1_*p*>0_1_*p*<1_	max_*i*,*j*_(*x*_*i*_ − *x*_*j*_)^2^	1 − ∏_*i*_ *x*_*i*_ − ∏_*i*_(1 − *x*_*i*_)

Another interpretation of heterozygosity is that it is twice the variance of the *x*_*i*_ over the population. This can be obtained by considering the random variable *x*_*i*_ where *i* is selected uniformly at random. This is a Bernoulli random variable with parameter *p*, which has variance *p*(1 − *p*), or half our heterozygosity score (see Table 1).

Now we consider extending the heterozygosity score to a collection of populations. Let *Y* = {*X*_*i*_|*i* = 1, …, *m*} be a set of populations, such that for all *i*, |*X*_*i*_| = *n*. We define *p*_*i*_ to be the fraction of 1s in population *X*_*i*_ at a given site and $\bar{p}=\frac{1}{m}\sum_{i=1}^{m}p_{i}$, the fraction of 1s over all the populations pooled together, assuming that all populations are of the same size. This assumption could readily be changed to account for populations of different sizes, with the appropriate weighting (see S2 Text).

**Fig 1:**
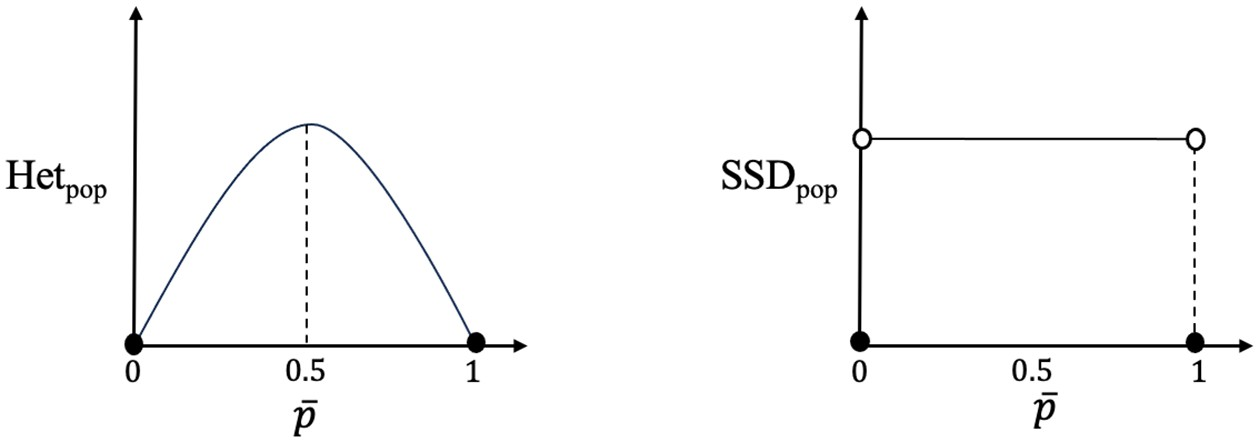
The values of Het_*pop*_ and SSD_*pop*_ as a function of $\bar{p}$ in the pooling approach.

Using the *pooling* approach (Het_pooling_), the Het score of a collection of populations is obtained by substituting *p* with $\bar{p}$ in the first expression for Het_ind_ in Table 1. It takes value 0 when $\bar{p}$ is 0 or 1, and takes a maximum value of 0.5 when $\bar{p}=0.5$ (see Fig 1 and Table 2). Effectively all population structure is ignored.

**Table 2:** Het scores and SSD scores defined on a single locus for a collection of populations. The index *i* ranges over the populations.

	Het_pop_	SSD_pop_
**Pooling**	$2\bar{p}(1-\bar{p})$	$1_{\bar{p}>0}1_{\bar{p}<1}$
**Averaging**	$\frac{1}{m}\Sigma_{i}2p_{i}\left(1-p_{i}\right)$	$\frac{1}{m}\Sigma_{i}1_{p_{i}>0}1_{p_{i}<1}$
**Differencing**	$\frac{1}{m^{2}}\Sigma_{i,j}{\left(p_{i}-p_{j}\right)}^{2}$	max_*i*,*j*_(*p*_*i*_ − *p*_*j*_)^2^
**Fixing**	$\frac{1}{m^{2}}\Sigma_{\begin{array}{c} i,j\\ i\neq j \end{array}}\left[1-p_{i}p_{j}-\left(1-p_{i}\right)\left(1-p_{j}\right)\right]$	1 − ∏_*i*_ *p*_*i*_ − ∏_*i*_(1 − *p*_*i*_)

The Het score of a collection of populations in the *averaging* approach (Het_averaging_) is obtained by substituting *p* with *p*_*i*_ in the expression 2*p*(1 − *p*) and averaging over all populations *i* (see Table 2). We can consider this as the within-population heterozygosity averaged over the collection of populations.

The Het score by the pairwise differencing approach (Het_differencing_) is obtained by substituting *x*_*i*_ in the second column of Table 1 with *p*_*i*_ (see Table 2). This is the between-population heterozygosity: if all the populations have the same value of *p*_*i*_, it is zero, regardless of that *p*_*i*_ and so the heterozygosity within populations. This score is double the variance of the *p*_*i*_ values (see S3 Text for the proof).

Lastly, the Het score in the fixing approach (Het_fixing_) is based on considering what happens in the long term if populations in the collection remain isolated from each other. In an isolated population with gene frequency *p*_*i*_, we expect over many generations the alleles will fix to either 0 or 1. Under simple models of neutral genetic drift, the probability of getting 1 is *p*_*i*_. After the process of fixing has occurred, we can then assume taking an individual randomly from each population to form a new population and applying our single-population measure of diversity to that population, giving a score. Then, we calculate the expected value over the future evolution of all populations. This can be obtained by substituting *x*_*i*_ with *p*_*i*_ in an expression for Het_ind_ which is linear in terms of *x*_*i*_ (shown in the last column of Table 1). The resulting expression is in the last row of Table 2.

Interestingly, all measures in the Het_pop_ column of Table 2 coincide with quantities in Caballero and Toro’s analysis [17] if we restrict their more general framework to the presence or absence of single allele per site (e.g. the SNP data we consider here). In their study, *GD*_*T*_ refers to the total genetic diversity and corresponds to our Het_pooling_. Their *GD*_*WS*_ is within-population genetic diversity and corresponds to our Het_averaging_, and their *GD*_*BS*_ is between-population genetic diversity which corresponds to our Het_differencing_. From their work, we see the relation *GD*_*T*_ = *GD*_*WS*_ + *GD*_*BS*_ or in our terminology Het_pooling_ = Het_averaging_ + Het_differencing_. Finally, ${\text{Het}}_{\text{fixing}}={\text{Het}}_{\text{pooling}}-\frac{1}{m}{\text{Het}}_{\text{averaging}}$, or equivalently $GD_{T}-\frac{1}{m}GD_{WS}$ (see S5 Text for more details).

All the formulas we have provided so far apply to a single locus. To apply to multiple loci we sum these formulas over all loci. Moreover, in the current formulations, we assume the populations are the same size; we have also extended the formulations to include population sizes (see S2 Text).

### Split system diversity: From individuals to populations

For any set *X* of individuals, split system diversity is a measure of diversity that can be calculated for any collection of splits of *X*, where a split is defined as a bipartition of *X* into two non-empty disjoint sets. When we have SNP data, we can define a collection of splits according to the presence or absence of a SNP at each locus. We define the SSD score of *X* to be

$$SSD\left(X\right)=\{\begin{array}{cc} 1 & \left(\exists i\;\text{s.t.}\;x_{i}=1\right)\;\text{and}\;\left(\exists j\;\text{s.t.}\;x_{j}=0\right),\\ 0 & \text{otherwise}. \end{array}$$

As with heterozygosity, we can express the SSD score in three equivalent expressions as shown in Table 1, where we maintain the same assumptions for *X* and *p* as previously specified. We can employ the four proposed methods to extend these definitions to populations.

As before, we first consider the case of a single locus, and describe the four measures of diversity for a collection of populations based on SSD. In the *pooling* approach, the SSD score of a set of populations is 1 if there exists at least one population with an individual in state 1 at the given locus and at least one population with an individual in state 0, and it is 0 otherwise (see Fig 1). In the *averaging* approach, however, the SSD score is the fraction of populations who have at least one individual in state 1 and one in state 0. The *pairwise differencing* measure is the maximum difference between the *p*_*i*_ squared. Finally, in the *fixing* approach the SSD score is obtained by replacing *x*_*i*_ with *p*_*i*_ in the last column of Table 1. It is the probability of obtaining both an individual with state 0 and an individual with state 1 if we select one individual from each population uniformly at random at some future time following fixation. See Table 2 for all of these values, which all lie between 0 and 1 for a single locus. The data and code used for the analyses in this study are publicly available at the following GitHub repository: https://github.com/nabhari/population-diversity-tool.

## Results

### Allele frequency distribution

**Fig 2:**
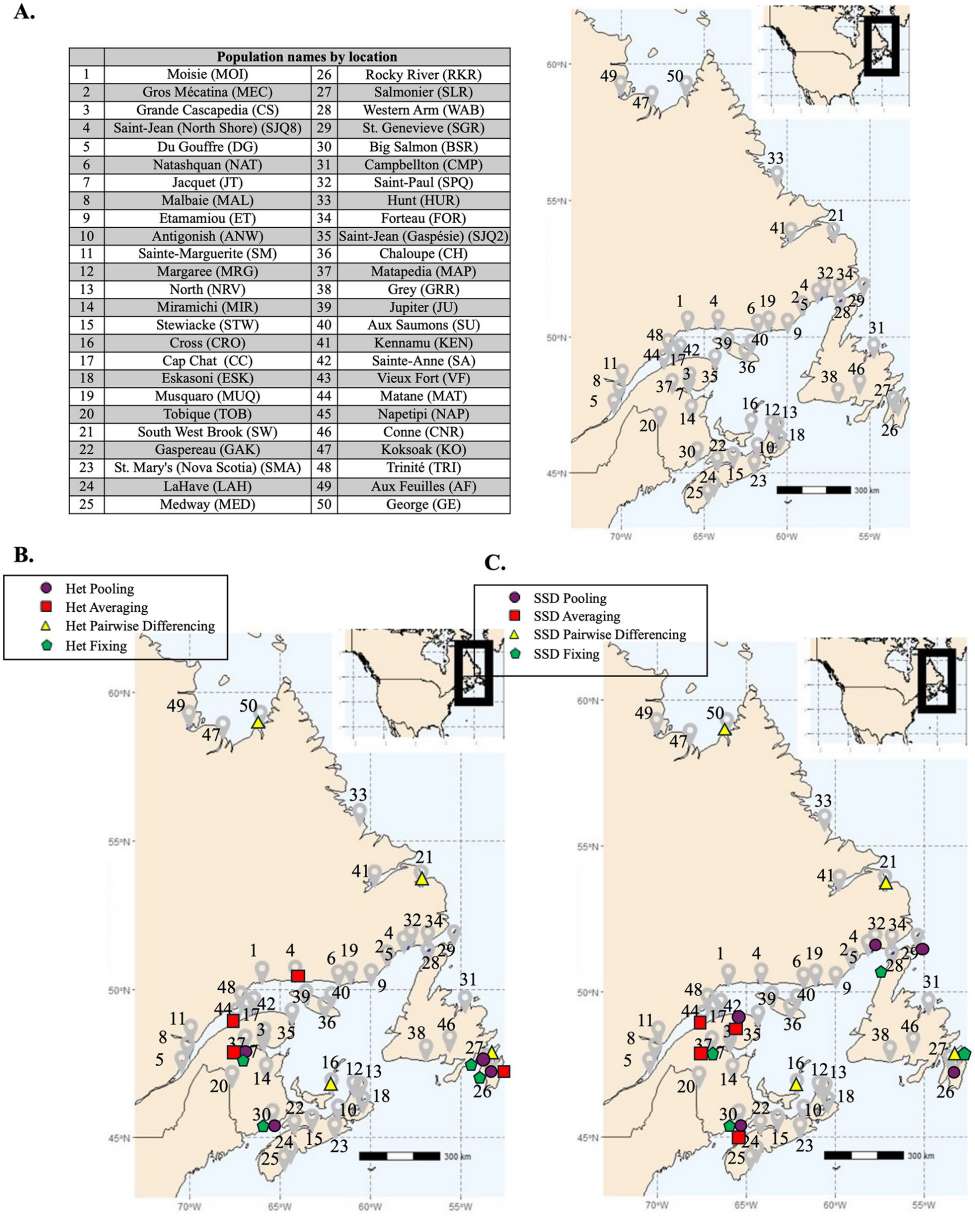
**A. Map of Atlantic salmon populations.** This map shows 50 sampling locations of Atlantic salmon in gray. The name of each population shown in the table on the left is based on the population’s location. **B.** Maximum-Diversity Sets (*k* = 4). Comparison of Het-based measures on the map. **C.** Maximum-Diversity Sets (*k* = 4). Comparison of SSD-based measures on the map. Note that there are two optimal sets by the pooling approach, {26, 30, 32, 42} and {29, 30, 32, 42}. Map data is provided by the maps package in R (https://cran.r-project.org/package=maps), which sources public domain data. The basemap is sourced from Natural Earth, public domain (https://www.naturalearthdata.com) and is compatible with the CC BY 4.0 License.

A total of 50 Canadian populations of Anadromous Atlantic salmon (*Salmo salar*), a species of significant conservation and management concern in North America, have been identified by Fisheries and Oceans Canada (DFO) [33]. A geographical representation of these populations is available in Fig 2A, illustrating their respective locations. In a dataset presented by J.S. Moore et al. [33, 34], 3192 SNPs were recorded, following standard filtering protocols, for between 8–25 individual salmon from each of these 50 populations (note that only the variant call format (VCF) files were available for the analysis in this paper). The principal objective of Moore et al. [33] was to find distinct conservation units, denoting identifiable and separate population clusters of the species to manage as independent entities, and to compare whether different subsets of markers denoted similar entities.

**Fig 3:**
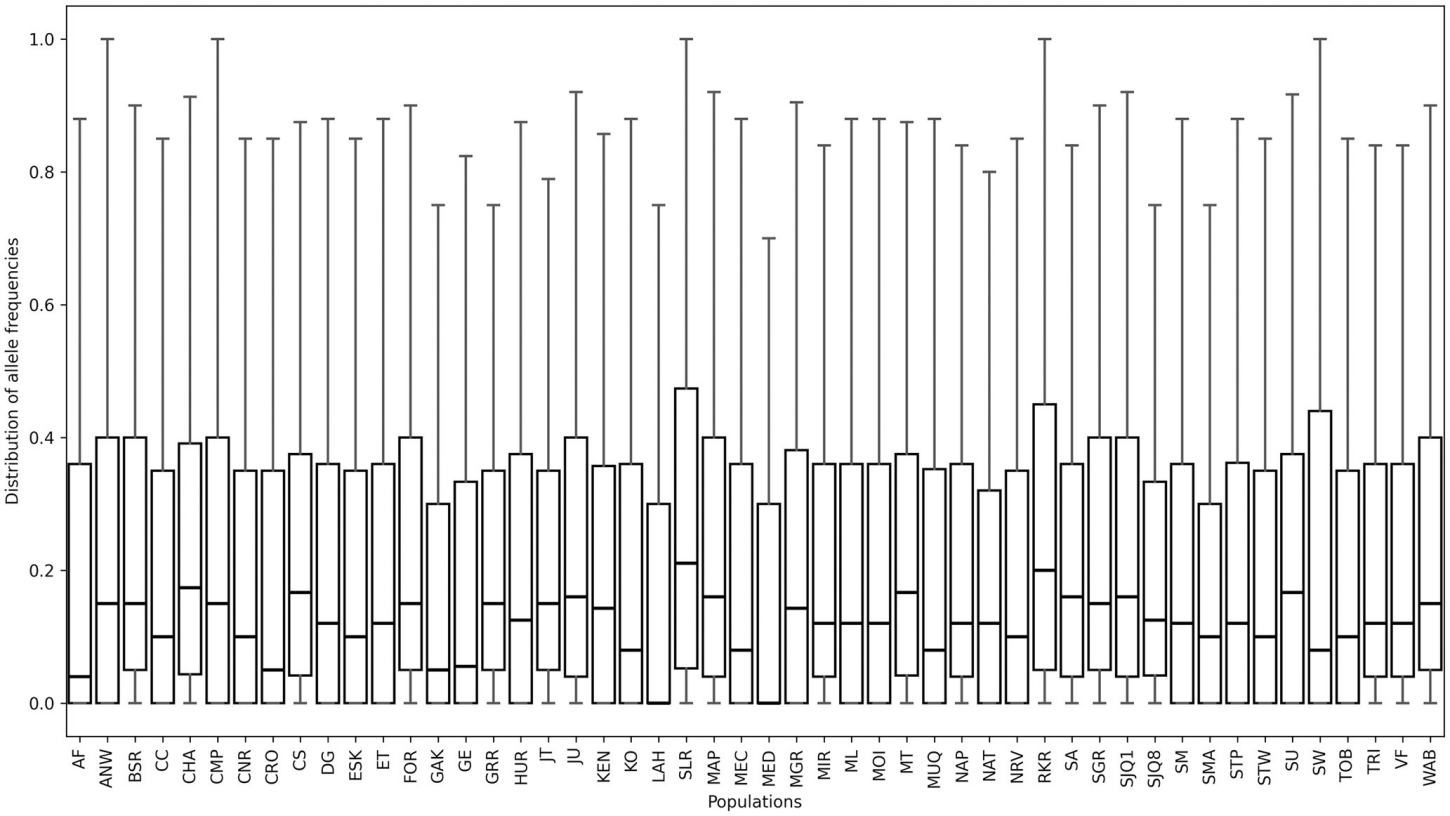
Distribution of allele frequency. Box plots illustrating the distribution of allele frequency values (*p*_*i*_) across 50 populations of Atlantic salmon. Each box corresponds to the variability of 3192 frequencies in one population, and the lines inside each plot indicate the median.

We used the SNP data of these 50 Atlantic salmon populations to investigate the correlations between our proposed population-level diversity measures. To apply our measures we first converted the SNP entries to minority allele presence-absence data, scoring each individual as either having the majority SNP (0) or any minority SNP (1). We then calculated the allele frequencies per population over all loci. This led to a 50 × 3192 matrix with each row representing a population and each column a locus. Each entry, *p*_*ij*_, in this matrix, is the minor allele frequency of population *i* at locus *j*. In this paper, however, we only use *p*_*i*_ to refer to the allele frequency of population *i* at a single locus as all the equations in Table 2 are defined for a single locus for simplicity. For most populations, the majority of sites have allele frequencies close to 0. This is expected given the scarcity of SNP occurrences within each population. Fig 3 shows the distribution of *p*_*i*_ values over all loci for each population with their respective median points.

### Correlation of Het-based diversity measures

The population level diversity measures based on heterozygosity in Table 2 were applied to all subsets of Atlantic salmon populations with sizes *k* = 2, 3, and 4. For each subset, we calculated the diversity per locus using the equations in Table 2. The diversity scores across all loci were summed to obtain the total diversity score for the subset. This process was repeated for all subsets of size *k* using each Het-based measure from Table 2. For each collection of *k*-size subsets, the Pearson correlation (*r*) between each pair of the Het-based measures was calculated (see Table 3). Here, we had to use a brute-force approach for the correlation analysis and for finding maximum diversity sets and so we are constrained to small values of *k* due to computational limitations. Later, we do the same analysis for larger *k* values but for a smaller randomly chosen sample of subsets.

**Table 3:** The Pearson correlation coefficients (*r*) of diversity measures based on heterozygosity and split system diversity applied on subsets of Atlantic salmon populations with size *k* = 2, 3, and 4.

Diversity Measures based on Heterozygosity	Het Pooling			Het Averaging			Het Pairwise Differencing		
Subset size	k = 2	k = 3	k = 4	k = 2	k = 3	k = 4	k = 2	k = 3	k = 4
Het Averaging	0.88	0.83	0.80	-	-	-	-	-	-
Het Pairwise Differencing	0.10	0.14	0.16	-0.39	-0.44	-0.46	-	-	-
Het Fixing	0.90	0.96	0.98	0.58	0.64	0.66	0.52	0.41	0.37
Diversity Measures based on Split System Diversity	SSD Pooling			SSD Averaging			SSD Pairwise Differencing		
Subset size	k = 2	k = 3	k = 4	k = 2	k = 3	k = 4	k = 2	k = 3	k = 4
SSD Averaging	0.89	0.81	0.75	-	-	-	-	-	-
SSD Pairwise Differencing	-0.10	-0.05	-0.01	-0.46	-0.52	-0.54	-	-	-
SSD Fixing	0.73	0.77	0.78	0.49	0.54	0.55	0.52	0.42	0.38

**Fig 4:**
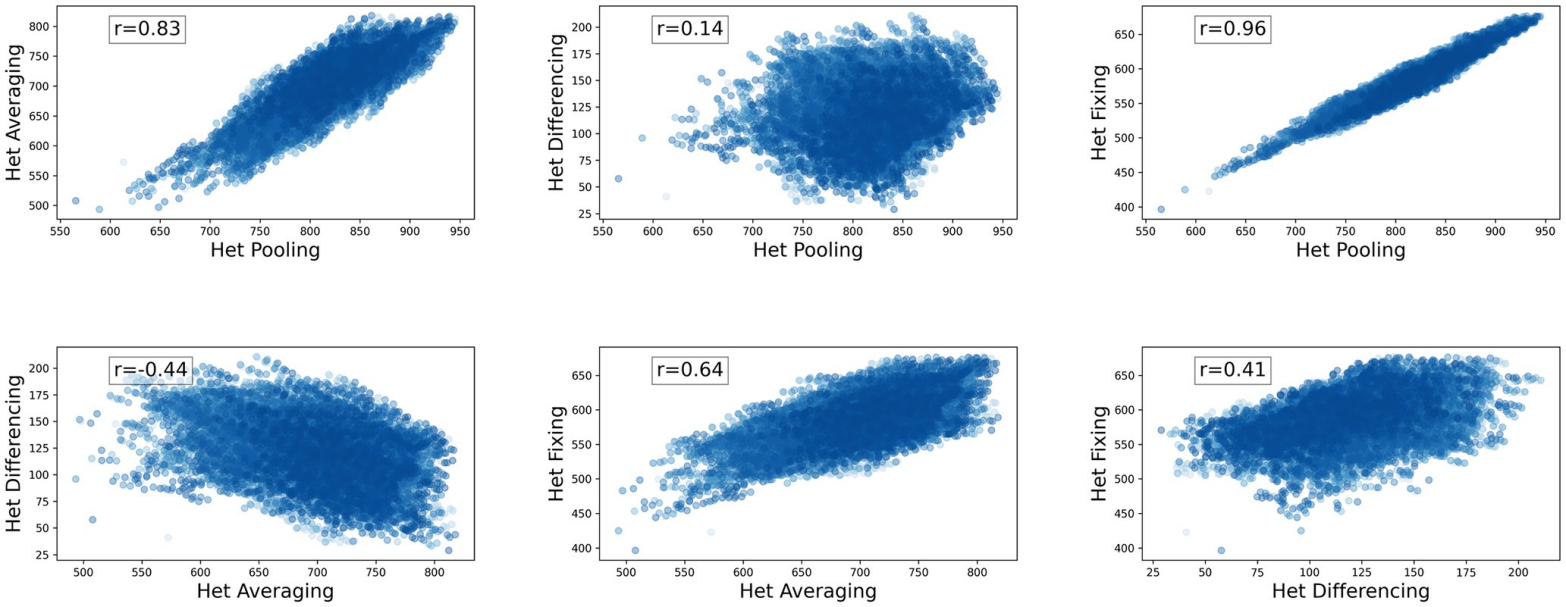
Correlation of diversity metrics based on heterozygosity in Atlantic salmon. Each subplot presents the correlation of two diversity functions measured on sets of populations with size 3. Each blue dot is a set of 3 populations. The *x* and *y* axes are all choices of the population diversity measures from Table 2.

As shown in Fig 4 and Table 3, Het_pooling_, Het_averaging_, and Het_fixing_ are highly correlated with each other. This makes sense because all tend to be increased by having *p*_*i*_ values closer to 0.5. We see a different pattern with Het_differencing_: it is positively correlated with Het_fixing_, but negatively correlated with Het_averaging_. This is also expected: Het_differencing_ increases when *p*_*i*_ values in different populations are different from each other, whereas Het_averaging_ increases when *p*_*i*_ values are closer to 0.5.

To investigate whether these correlations apply only to salmon SNP data or more broadly, we performed the same analysis on two simulated data sets. First, we calculated the correlations between all Het-based diversity measures on all collections of populations of size 3 for the Atlantic salmon data. Then, we randomly permuted the elements of the matrix of *p*_*i*_ values across all populations and all loci (each row in the matrix corresponds to a population and each column is the *p*_*i*_ at a locus). We obtained the analogous correlation results for this artificial data set (see S2 Fig for the correlation plots). We observe that qualitative features are the same; if two measures are positively correlated in the salmon data, they are positively correlated in the randomized data. With few exceptions, the magnitudes of correlation are similar. This indicates that the patterns we observe are not due to any particular population structure in the salmon data, but due to the nature of our measures, and possibly the fact that most of our *p*_*i*_ are close to zero.

To test this second possibility, we generated a similar set of synthetic data, but this time selecting every *p*_*i*_ uniformly from [0, 1]. Again, a similar pattern of correlation holds, supporting that we expect to see such correlations in a broad variety of biological examples (see S3 Fig for the correlation plots).

We then used a brute-force search to compute the maximum diversity sets and scores for salmon populations with sizes *k* = 2, 3, and 4. The results, presented in Table 4 (the optimal population locations are shown on the map for *k* = 4 in Fig 2B), indicate that the measures generally disagree on the maximum diversity subset of a given size. The exception is that Het_pooling_ and Het_fixing_ agree on maximum diversity subsets for *k* = 3 and *k* = 4. Given the correlations we observed among the measures in the Atlantic salmon data, we might expect maximum diversity sets to capture similar amounts of diversity even if the sets are different. For example, if we consider the Het_pooling_ versus Het_averaging_ correlation plot in Fig 4 (top left), the points on the top right corner maximize both axes but could be two different sets. On the other hand, if two measures are not highly correlated (e.g. Het_pooling_ and Het_differencing_), we would expect they lead to quite different maximum diversity sets because these maximize diversity measures that are defined from two distinctly different points of view.

**Table 4:** Maximum diversity set(s) of size *k* = 2, 3, 4 and scores obtained by applying each of the Het-based and SSD-based measures on Atlantic salmon populations. The numbers in each set correspond to the population locations as indicated in Fig 2A.

Diversity Measures based on Heterozygosity and Split System Diversity	*k* = 2		*k* = 3		*k* = 4	
Optimal solutions	Set	Score	Set	Score	Set	Score
Het Pooling	{26, 37}	468.50	{27, 30, 37}	472.68	{26, 27, 30, 37}	476.75
Het Averaging	{37, 44}	819.34	{4, 37, 44}	817.90	{4, 26, 37, 44}	816.10
Het Pairwise Differencing	{16, 50}	81.14	{16, 27, 50}	105.39	{16, 21, 27, 50}	111.68
Het Fixing	{27, 37}	532.84	{27, 30, 37}	677.01	{26, 27, 30, 37}	751.90
SSD Pooling	{26, 37}	3001	{30, 32, 42}	3103	{26/29, 30, 32, 42}	3152
SSD Averaging	{30, 37}	2641.5	{30, 37, 44}	2627	{30, 37, 42, 44}	2619.5
SSD Pairwise Differencing	{16, 50}	324.57	{16, 27, 50}	554.02	{16, 21, 27, 50}	692.15
SSD Fixing	{27, 37}	1065.69	{27, 30, 37}	1532.27	{27, 28, 30, 37}	1817.30

### Correlation of SSD-based diversity measures

We applied population-level diversity measures based on SSD, as outlined in Table 2, to all subsets of Atlantic salmon populations, with sizes *k* = 2, 3, and 4. For each subset, we calculated the diversity per locus using the equations in Table 2. The diversity scores across all loci were summed to obtain the total diversity score for the subset. This process was repeated for all subsets of size *k* using each SSD-based measure. Then for every pair of measures, we calculated the Pearson correlation (*r*) among the SSD-based measures, and the results are summarized in Table 3. Similar to the Het-based measures analysis, SSD-based analysis was also done for larger *k* values across random subsets, which we discuss later. Additionally, we conducted an exhaustive search to identify the maximum SSD set(s) of salmon populations for different values of *k* using each of the SSD-based metrics (Table 4). Similar to Het-based measures, here we expect that highly correlated measures result in maximum diversity sets that are close in terms of diversity but not necessarily equal, and measures that are poorly correlated are expected to produce different maximum diversity sets.

**Fig 5:**
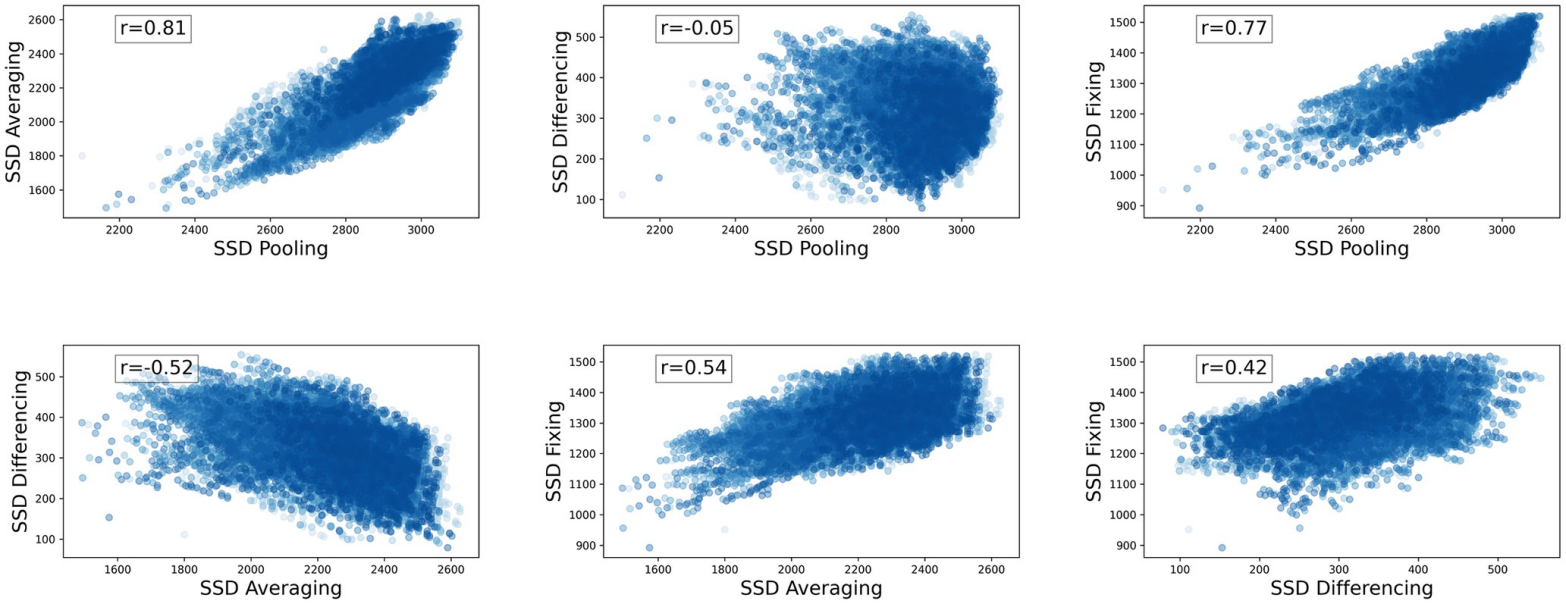
Correlation of diversity metrics based on SSD in Atlantic salmon. Each subplot presents the correlation of two diversity functions measured on sets of populations with size 3. Each blue dot is a set of 3 populations. The *x* and *y* axes are all choices of the population diversity measures from Table 2.

Given the close relationship between SSD and heterozygosity, we expected these measures to exhibit similar correlation patterns for salmon subsets with a size of *k*. Notably, in Fig 5, the scatter plot illustrating SSD_fixing_ and SSD_differencing_ is very similar to the Het-based plot for the same pair of measures with similar correlations. On the other hand, the maximum diversity sets found by each of the SSD measures are moderately different from those found by Het measures, as expected, in the sense that was explained earlier about the maximum diversity sets of highly correlated measures (see Table 4). To further understand the relationship between Het-based and SSD measures in population diversity assessment, we conducted a correlation analysis between these two categories of diversities.

### Heterzygosity versus split system diversity

**Fig 6:**
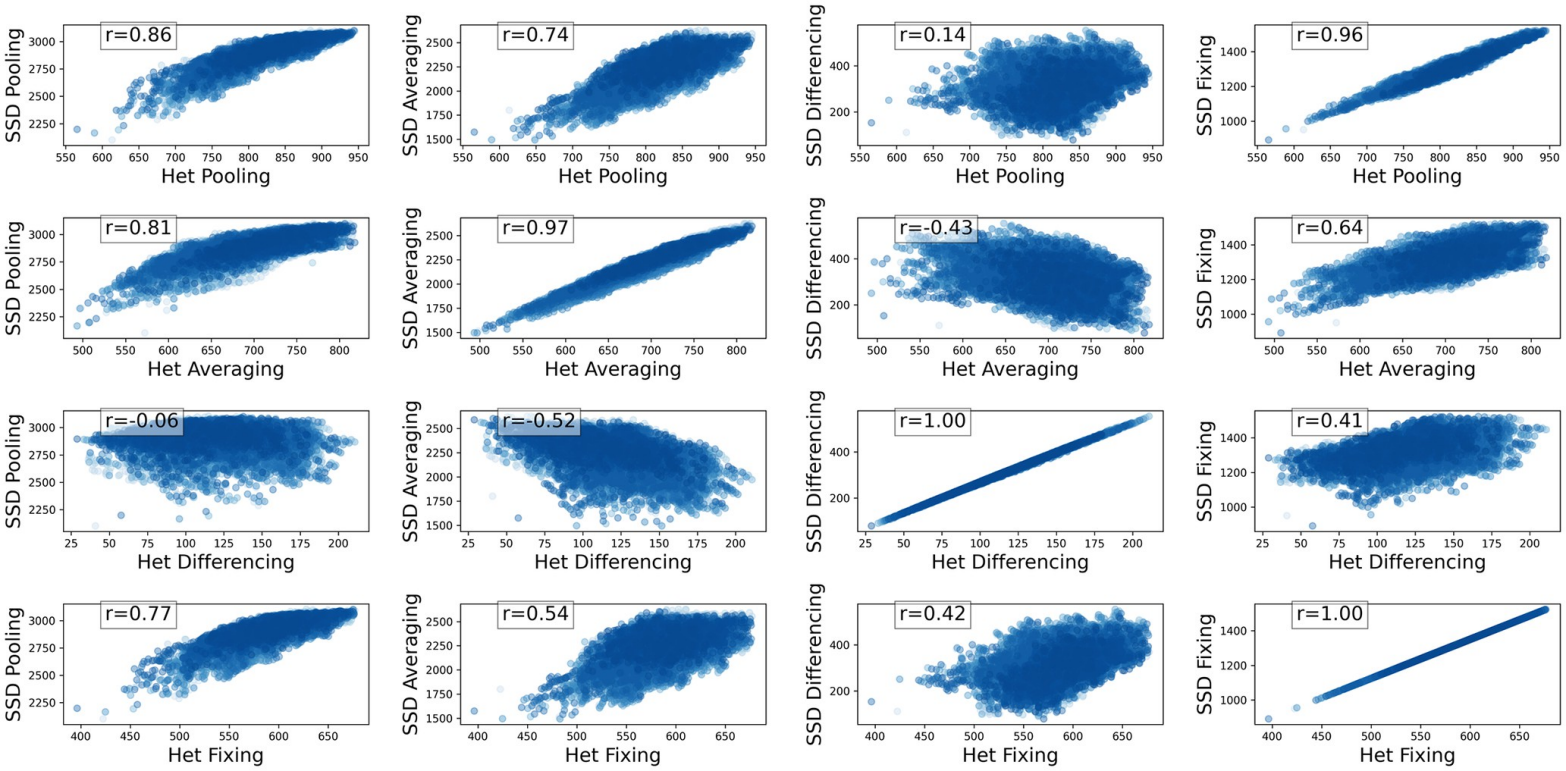
Correlation of diversity metrics based on SSD and Het in Atlantic salmon. Each subplot presents the correlation of two diversity functions measured on sets of populations with size 3. Each blue dot is a set of 3 populations. The *x* and *y* axes are all combinations of the population diversity measures from Table 2.

The results shown in Table 5 indicate that for each strategy (i.e. pooling, averaging, pairwise differencing, or fixing), the Het version and the SSD version are highly correlated (Fig 6). Similar to the previous sections, all of our population-level diversity measures are a sum of a diversity measure for a single locus over all loci. Assuming independence between loci, if we explain a high correlation between two measures at one locus, we have explained it for the summation over all loci (proof in S1 Text).

**Table 5:** The Pearson correlation coefficients (*r*) of Het-based and SSD-based diversity measures applied on subsets of Atlantic salmon populations with size *k* = 2, 3, and 4.

Diversity Measures	SSD Pooling			SSD Averaging			SSD Pairwise Differencing			SSD Fixing		
Subset size	k = 2	k = 3	k = 4	k = 2	k = 3	k = 4	k = 2	k = 3	k = 4	k = 2	k = 3	k = 4
Het Pooling	0.90	0.86	0.83	0.81	0.74	0.71	0.10	0.14	0.17	0.90	0.96	0.98
Het Averaging	0.88	0.81	0.76	0.97	0.97	0.97	-0.39	-0.43	-0.45	0.58	0.64	0.65
Het Pairwise Differencing	-0.10	-0.06	-0.02	-0.46	-0.52	-0.55	1.00	1.00	1.00	0.52	0.41	0.37
Het Fixing	0.73	0.77	0.77	0.49	0.54	0.55	0.52	0.42	0.37	1.00	1.00	1.00

To explore the striking correlation between the SSD and Het measures, we permuted the *p*_*i*_ values from the salmon data to get a dataset with the same size and distribution of *p*_*i*_ values but no correlations between loci or populations. In this dataset, the correlations between corresponding diversity measures (e.g. between Het_fixing_ and SSD_fixing_, or Het_differencing_ and SSD_differencing_) were still high (see the Het versus SSD correlation plots in S2 Fig). Next, we checked the correlation in a dataset with *p*_*i*_ values selected independently and uniformly at random in [0, 1]. The correlations were still very high. With these experiments, we can conclude that the correlations between Het-based and SSD-based measures of the same type are likely to be high regardless of the distribution of allele frequencies. We conjecture that they will be roughly interchangeable as measures of population diversity in many situations of conservation importance. Specifically, it can be proven that the correlation between Het_fixing_ and SSD_fixing_ for sets of populations with sizes *k* = 2, and 3 is exactly 1 and starts decreasing gradually for *k* ≥ 4 (see S4 Text for the proof).

### Correlation trends and larger *k* values

**Fig 7:**
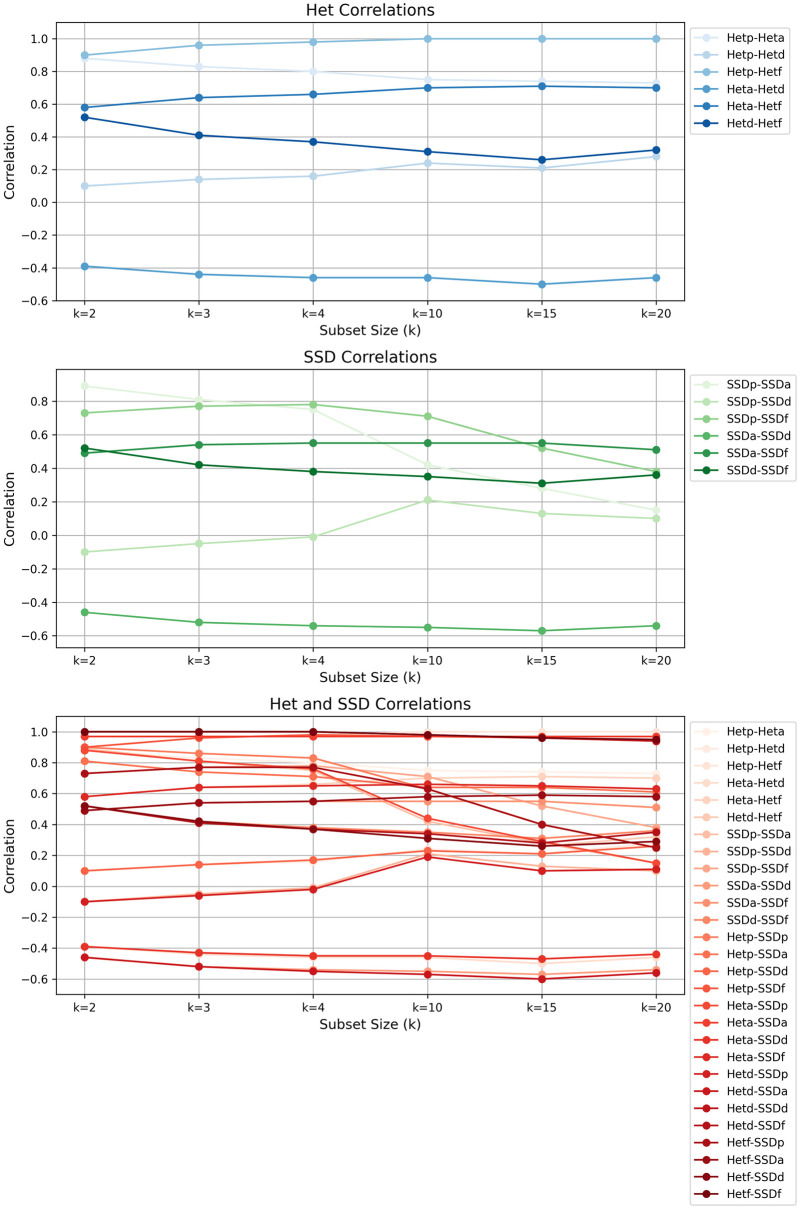
Trends of correlations between diversity metrics based on SSD and Het in Atlantic salmon with respect to *k*. Each subplot shows the correlation trends for *k* = 2, 3, 4, 10, 15, 20 for two diversity functions, as indicated in the legend. The correlation trends for Het-based, SSD-based, and Het versus SSD measures are shown in blue, green, and red, respectively. In the figure legend, ‘-p’, ‘-a’, ‘-d’, and ‘-f’ correspond to Pooling, Averaging, Differencing, and Fixing, respectively.

Given that $\left(\begin{array}{c} n\\ k \end{array}\right)$ rapidly grows for large *k* values (up to $k=\frac{n}{2}$), it is computationally expensive to calculate all measures in Table 2 for every subset of size *k* of Atlantic salmon populations for correlation analysis. To manage this, we randomly sampled 1000 subsets of size *k* for *k* = 10, 15 and 20, calculated the population-level diversity measures from Table 2 as previously described, and computed pairwise correlations for those random samples. In Fig 7, we show the correlations for different values of *k*, for every pair of diversity measures. We find that the correlations for smaller *k* values are indicative of the correlations for large *k* values.

To ensure the accuracy of the correlations we report for larger *k* values, which are based on a sample, we calculate the standard error of each correlation (*r*) between pairs of measures for a given *k*. We calculated the standard errors using the formula $\frac{\left(1-r^{2}\right)}{\sqrt{N-3}}$, as recommended in [35] for computing the standard error of Pearson correlations, where *r* is the Pearson’s correlation coefficient and *N* = 1000 is the number of observations. We found that all standard errors were below 0.03. We repeated this experiment with another random sample of 1000 subsets of size *k* = 10, 15 and 20 with a different seed, and the distributions of standard errors from the two samples were very similar (See S4 Fig). From this experiment, we can conclude that the correlations we observed for larger *k* values are reliable because of the low standard errors and consistent results across two different random samples.

## Dryad DOI

10.5061/dryad.sb601 [34].

## Discussion

Given a diversity measure for a single population, we proposed four approaches for extending it to measure the diversity of a collection of populations: pooling, averaging, pairwise differencing, and fixing. These approaches provide distinct perspectives on diversity assessment. While our focus remains on conservation, these methods can also be used in other fields.

In the example of the islands (A and B) that we referred to in the introduction, each with two populations with respective allele frequencies of A=(0.1, 0.9) and B=(0.5, 0.5), not all of our proposed measures agree on which island to preserve. If we use heterozygosity, the pooling approach does not differentiate between the two islands and would give both the same score of 0.5—here we treat a collection of populations as one large population on each island. However, in the averaging approach, island B would be accorded a higher priority (with a score of 0.5) compared to island A (with a score of 0.18) as the allele frequencies in each population are taken into account for measuring diversity. On the other hand, the pairwise differencing and the fixing approaches would both agree that island A is more important for conservation. If we change allele frequencies for islands A and B to (0.2, 0.2) and (0.1, 0), respectively, then a different pattern emerges. Now pooling, averaging, and fixing all rate island A as having higher diversity, whereas pairwise differencing gives a higher score to island B.

This latter example is the pattern that we observe in the salmon data. Recall that we observed Het_pooling_, Het_fixing_, and Het_averaging_ to co-vary, though to varying degrees (with Het_averaging_ exhibiting a slightly lower degree of similarity with the others). However, Het_differencing_ produces notably distinct results. As in the second version of our island example, this difference arises from the fact that the first three measures (Het_pooling_, Het_fixing_, and Het_averaging_) aim for populations with *p*_*i*_ values closer to 0.5 without considering differences between them, while Het_differencing_ focuses on the differences between population-level *p*_*i*_ values. This becomes evident in the context of the salmon data where *p*_*i*_s are close to 0, leading Het_differencing_ to yield a distinct result. We conjecture that the observed pattern in the salmon data differs from that in the first island example because the *p*_*i*_ values used in that example (such as 0.5 and 0.9) are uncommon in the allele frequencies of the Atlantic salmon data.

A substantial limitation of our work is that, especially for rare loci, the *p*_*i*_ values may not be well known from the sample (see [36]). We have not incorporated uncertainty in the *p*_*i*_ values here. We would expect that averaging the measures over many loci helps to overcome issues related to this uncertainty but more work is warranted.

The salmon data and the toy examples demonstrate that the population diversity measures capture diversity from varied perspectives but only sometimes agree on the optimal sets for prioritization. This highlights the importance of the definition used to measure population-level diversity for conservation purposes, or indeed any scenario where identifying optimal sets is crucial. In our proposed approaches, we may opt for Het_pooling_ to conserve overall heterozygosity, particularly in scenarios where significant gene flow is expected in the future. Alternatively, Het_averaging_ could be employed if populations that are individually heterozygous have a higher priority for conservation. For preserving populations with maximum divergence, such as those in remote regions exhibiting local adaptation, Het_differencing_ would be suitable. Finally, if the aim is to conserve total heterozygosity post-drift-induced fixation within each population, Het_fixing_ would be an appropriate choice.

## Supporting information

S1 Text Correlations and summation over all loci. This section explains how the correlation of the sum over all loci for pairs of measures is the same as the correlation of the individual loci.
(PDF)

S2 Text Generalization to a set of populations of different sizes. This section generalizes the formulas for heterozygosity (*Het*) to populations of varying sizes.
(PDF)

S3 Text Het_differencing_ and variance of *p*_*i*_. This section explains that the Het_differencing_ score is equivalent to twice the variance of the *p*_*i*_ values.
(PDF)

S4 Text Perfect correlations between Het_fixing_ and SSD_fixing_. This section explains why the correlations between Het_fixing_ and SSD_fixing_ are almost perfect.
(PDF)

S5 Text The relation between Het_fixing_, Het_pooling_, and Het_averaging_. This section explains how the formulations for Het_fixing_, Het_pooling_, and Het_averaging_ are related.
(PDF)

S1 Fig Correlation of diversity metrics based on SSD and Het in Atlantic salmon. Each subplot presents the correlation of two diversity functions measured on sets of populations with size 3. Each blue dot is a set of 3 populations. The *x* and *y* axes are all combinations of the population diversity measures based on Het and SSD.
(PDF)

S2 Fig Correlation of diversity metrics based on Het and SSD on a randomly permuted version of the Atlantic salmon data. Each subplot presents the correlation of two diversity functions measured on sets of populations with size 3. Each orange dot is a set of 3 populations. The *x* and *y* axes are all combinations of the population diversity measures based on Het and SSD.
(PDF)

S3 Fig Correlation of diversity metrics based on SSD and Het in a randomly generated dataset with 50 populations and 3192 loci, where *p*_*i*_ values are uniformly sampled from [0, 1]. Each subplot presents the correlation of two diversity functions measured on sets of populations with size 3. Each purple dot is a set of 3 populations. The *x* and *y* axes are all combinations of the population diversity measures based on Het and SSD.
(PDF)

S4 Fig Standard error of the correlations of diversity metrics for *k* = 10, 15 and 20. Each subplot shows the distribution of the standard errors of the correlations between all pairs of diversity measures in Table 2, which are computed for 1000 randomly selected subsets of size k. The distribution in red and purple corresponds to two random samples of size 1000 with different seeds.
(PDF)

S1 Codes Python code for calculating diversity measures and correlations. This file contains Python scripts, available in this repository, https://github.com/nabhari/population-diversity-tool, used for calculating population-based diversity measures, including Het-based and SSD-based metrics, as well as performing correlation analyses between them. The code also includes modules for brut-force search and plotting correlation results.
(ZIP)
