# Supplementary material for: Measuring genetic diversity across populations
Source: PLoS Comput Biol. 2024 Dec 4;20(12):e1012651. doi: 10.1371/journal.pcbi.1012651 (PMC11649088; doi:10.1371/journal.pcbi.1012651)
Supplement: S1 Text — This section explains how the correlation of the sum over all loci for pairs of measures is the same as the correlation of the individual loci. (PDF) [file pcbi.1012651.s001.pdf]

S1. Text. Correlations and summation over all loci

Let X_i^α and X_i^β be the α and β measures of diversity of a set of populations at locus i, $i = 1, \ldots, \ell$. If we randomly select populations, these are random variables. We assume that all X_i^α are i.i.d. and likewise for X_i^β. (This is only an approximation, but it is likely to be a good one when the total number of subpopulations is much greater than the number in our subsets.) Let $X^\alpha = \sum_i X_i^\alpha$, $X^\beta = \sum_i X_i^\beta$ be the sum over all loci.

Let X_i^α, X_i^β have means μ_α, μ_β and variances σ_α^2, σ_β^2. Then the mean of X^α and X^β are $\ell\mu_\alpha$ and $\ell\mu_\beta$, and the variances are $\ell\sigma_\alpha^2$ and $\ell\sigma_\beta^2$, by the properties of sums of independent random variables.

$$\begin{aligned}
\mathrm{corr}(X^\alpha, X^\beta) &= \frac{\mathbb{E}((X^\alpha - \ell\mu_\alpha)(X^\beta - \ell\mu_\beta))}{\ell\sigma_\alpha\sigma_\beta} \\
&= \frac{\mathbb{E}((\sum_i (X_i^\alpha - \mu_\alpha))(\sum_j (X_j^\beta - \mu_\beta)))}{\ell\sigma_\alpha\sigma_\beta} \\
&= \frac{\sum_i \mathbb{E}(X_i^\alpha - \mu_\alpha)(X_i^\beta - \mu_\beta)}{\ell\sigma_\alpha\sigma_\beta} \\
&= \frac{\ell\mathbb{E}(X_i^\alpha - \mu_\alpha)(X_i^\beta - \mu_\beta)}{\ell\sigma_\alpha\sigma_\beta} \\
&= \mathrm{corr}(X_i^\alpha, X_i^\beta)
\end{aligned}$$

So the correlation of the sum is the same as the correlation of the individual loci. Using the sum over loci in some SSD-based measures, such as $\mathrm{SSD}_{\mathrm{pooling}}$, directly corresponds to the number of columns that contain both states 0 and 1 for any given set of populations. We found it more practical to interpret the results using the sum rather than the average, as the latter may not yield an integer (e.g., 10.5), which doesn't clearly indicate how many loci with both states are present in the set. Additionally, the correlation values remain unchanged whether the sum or average is used, as multiplying the scores by a constant does not affect the results.
