# Supplementary material for: Measuring genetic diversity across populations
Source: PLoS Comput Biol. 2024 Dec 4;20(12):e1012651. doi: 10.1371/journal.pcbi.1012651 (PMC11649088; doi:10.1371/journal.pcbi.1012651)
Supplement: S2 Text — This section generalizes the formulas for heterozygosity (Het) to populations of varying sizes. (PDF) [file pcbi.1012651.s002.pdf]

S2 Text. Generalization to a set of populations of different sizes

We consider the case of one locus, where the value is either 0 or 1. Suppose we have m subpopulations, with the ith population having n_i individuals, and having p_i as the fraction of 1s at the locus.

The fraction of 1s in the total population is given by

$$\overline{p} = \frac{\sum_i n_i p_i}{\sum_i n_i} = \frac{1}{n} \sum_i n_i p_i,$$

since $\sum_i n_i p_i$ is the total number of individuals with a 1 at the locus, and $n = \sum_i n_i$ is the total number of individuals.

Accordingly, $\text{Het}_{\text{pooling}}$ is given by

$$\text{Het}_{\text{pooling}} = 2\overline{p}(1 - \overline{p})$$

as usual, with this appropriate definition of $\overline{p}$.

We define $\text{Het}_{\text{averaging}}$ for this situation as

$$\text{Het}_{\text{averaging}} = \frac{1}{n} \sum_i n_i 2p_i(1 - p_i)$$

which is the average of the heterozygosity in each population, weighted by the size of the population.

Finally, we define $\text{Het}_{\text{differencing}}$ for this situation as

$$\text{Het}_{\text{differencing}} = \frac{1}{n^2} \sum_{ij} n_i n_j (p_i - p_j)^2$$

Note that if we replace n_i with n/m then we obtain the expressions in the paper.

(The formula for $\text{Het}_{\text{fixing}}$ in terms of p_i does not change, since it does not depend on the the sizes of the individual populations.)

We now expand each of these expressions so that we can verify that $\text{Het}_{\text{pooling}} = \text{Het}_{\text{averaging}} + \text{Het}_{\text{differencing}}$.

$$
\begin{aligned}
\text{Het}_{\text{pooling}} &= 2\bar{p}(1-\bar{p}) \\
&= 2\left[\frac{1}{n}\sum_i n_i p_i\right]\left[\frac{1}{n}\sum_j n_j - n_j p_j\right] \\
&= \frac{2}{n^2}\sum_{i,j} n_i n_j p_i - \frac{2}{n^2}\sum_{i,j} n_i n_j p_i p_j \\
&= \frac{2}{n}\sum_i n_i p_i - \frac{2}{n^2}\sum_{i,j} n_i n_j p_i p_j
\end{aligned}
$$

$$
\begin{aligned}
\text{Het}_{\text{averaging}} &= \frac{2}{n}\sum_i n_i p_i(1-p_i) \\
&= \frac{2}{n}\sum_i n_i p_i - \frac{2}{n}\sum_i n_i p_i^2.
\end{aligned}
$$

$$
\begin{aligned}
\text{Het}_{\text{differencing}} &= \frac{1}{n^2}\sum_{i,j} n_i n_j (p_i - p_j)^2 \\
&= \frac{1}{n}\sum_i n_i p_i^2 - 2\frac{1}{n^2}\sum_{i,j} n_i n_j p_i p_j + \frac{1}{n}\sum_j n_j p_j^2 \\
&= \frac{2}{n}\sum_i n_i p_i^2 - \frac{2}{n^2}\sum_{i,j} n_i n_j p_i p_j
\end{aligned}
$$

Observe by cancelling terms that $\text{Het}_{\text{pooling}} = \text{Het}_{\text{averaging}} + \text{Het}_{\text{differencing}}$.
