# Supplementary material for: Measuring genetic diversity across populations
Source: PLoS Comput Biol. 2024 Dec 4;20(12):e1012651. doi: 10.1371/journal.pcbi.1012651 (PMC11649088; doi:10.1371/journal.pcbi.1012651)
Supplement: S3 Text — This section explains that the Hetdifferencing score is equivalent to twice the variance of the pi values. (PDF) [file pcbi.1012651.s003.pdf]

S3 Text. $\mathrm{Het}_{\mathrm{differencing}}$ and variance of p_i

We see that $\mathrm{Het}_{\mathrm{differencing}}$ is twice the variance of the p_i values in the following way:

$$\begin{aligned}\mathrm{Het}_{\mathrm{differencing}} = \frac{1}{n^2}\Sigma_{i,j}(p_i - p_j)^2 &= \frac{1}{n^2}[n\Sigma_i p_i^2 + n\Sigma_j p_j^2 - 2\Sigma_{i,j}p_i p_j] \\ &= \frac{1}{n^2}[2n\Sigma_i p_i^2 - 2n^2\bar{p}^2] \\ &= \frac{2}{n}\Sigma_i p_i^2 - 2\bar{p}^2 \\ &= 2[\frac{1}{n}\Sigma_i p_i^2 - \bar{p}^2]\end{aligned}$$
