# Supplementary material for: Measuring genetic diversity across populations
Source: PLoS Comput Biol. 2024 Dec 4;20(12):e1012651. doi: 10.1371/journal.pcbi.1012651 (PMC11649088; doi:10.1371/journal.pcbi.1012651)
Supplement: S4 Text — This section explains why the correlations between Hetfixing and SSDfixing are almost perfect. (PDF) [file pcbi.1012651.s004.pdf]

S4 Text. Perfect correlations between $\text{Het}_{\text{fixing}}$ and $\text{SSD}_{\text{fixing}}$

We observe that the correlation between $\text{Het}_{\text{fixing}}$ and $\text{SSD}_{\text{fixing}}$ is exactly 1 for two or three subpopulations ($k = 2$ or 3). Here we prove this by establishing the linear relation between these measures in this case.

We consider a single locus since if they are perfectly correlated at each locus, they will be perfectly correlated when we take the sum over all loci.

Recall that for a given single-population measure, the corresponding fixing measure is obtained by selecting an individual uniformly at random from each subpopulation and then applying the single-population measure to this collection. So when comparing $\text{Het}_{\text{fixing}}$ and $\text{SSD}_{\text{fixing}}$, we can first hold constant the particular alleles we get when selected from the subpopulations and then look at what the two different measures Het and SSD give.

First, let's consider the $k = 2$ case. We show the four possibilities for what individuals we draw from the populations and the resulting Het and SSD scores in the following table.

Alleles	Probability	Het	SSD
0 0	$(1 - p_1)(1 - p_2)$	0	0
0 1	$(1 - p_1)p_2$	1/2	1
1 0	$p_1(1 - p_2)$	1/2	1
1 1	$p_1 p_2$	0	0

The actual $\text{Het}_{\text{fixing}}$ and $\text{SSD}_{\text{fixing}}$ scores are obtained by averaging the values in the matching column in the table weighted by the probabilities in the second column. But since the Het is always half of SSD, $\text{SSD}_{\text{fixing}}$ is always twice $\text{Het}_{\text{fixing}}$, regardless of the values of p_1 and p_2. So for $k = 2$, $\text{Het}_{\text{fixing}}$ and $\text{SSD}_{\text{fixing}}$ always have a correlation of 1 when we sample from a larger set of populations.

For $k = 3$, the corresponding table (omitting the column of probabilities) is

Alleles	Het	SSD
0 0 0	0	0
0 0 1	4/9	1
0 1 0	4/9	1
0 1 1	4/9	1
1 0 0	4/9	1
1 0 1	4/9	1
1 1 0	4/9	1
1 1 1	0	0

To determine the Het values in the above table, in each of the cases where not all the alleles are the same, there are two of one type and one of another. So the probability of selecting two different ones at random is $1 - (1/3)^2 - (2/3)^2 = 4/9$. In each case Het is 4/9 times SSD so $\text{Het}_{\text{fixing}} = (4/9)\text{SSD}_{\text{fixing}}$.

When $k = 4$ the pattern breaks down. Below is the table for $k = 4$, but we only show a representative selection of rows.

Alleles	Het	SSD
0 0 0 0	0	0
0 0 0 1	3/8	1
0 0 1 1	1/2	1
0 1 1 1	3/8	1
1 1 1 1	0	0

Since the ratio between Het and SSD is not the same for all selections of alleles, we do not expect the correlation to be exactly 1 in this case.
