# Supplementary material for: Measuring genetic diversity across populations
Source: PLoS Comput Biol. 2024 Dec 4;20(12):e1012651. doi: 10.1371/journal.pcbi.1012651 (PMC11649088; doi:10.1371/journal.pcbi.1012651)
Supplement: S5 Text — This section explains how the formulations for Hetfixing, Hetpooling, and Hetaveraging are related. (PDF) [file pcbi.1012651.s005.pdf]

S5 Text. The relation between $\text{Het}_{\text{fixing}}$, $\text{Het}_{\text{pooling}}$, and $\text{Het}_{\text{averaging}}$

We recall that $\text{Het}_{\text{pooling}}$ is the overall heterozygosity of all individuals, and so is equal to the probability of two individuals being selected having different alleles, where we select each individual uniformly at random from all individuals, with replacement. We can alternatively think of this as, for each individual, selecting a population uniformly at random, and then selecting an individual uniformly at random from that population.

Now let us compute $\text{Het}_{\text{pooling}}$ differently, by conditioning on whether the two populations selected are the same. With probability 1/m, the two chosen populations are the same. If so, then the two individuals will be from the same population. If the population they are in is the ith, then the probability they have different alleles is $2p_i(1-p_i)$. More generally, we average over i to get that the probability of being different is $\text{Het}_{\text{averaging}}$. On the other hand, with probability $(m-1)/m$, we get different populations. Let us call the probability we get different alleles in this case h. So conditioning gives us

$$\text{Het}_{\text{pooling}} = \frac{1}{m}\text{Het}_{\text{averaging}} + \frac{(m-1)}{m}h$$

Recall that $\text{Het}_{\text{fixing}}$ is the heterozygosity of a population formed by selecting an individual at random from each subpopulation. So it is the same as selecting a population at random, then selecting an individual from it, selecting a population and selecting an individual from it, but with the requirement that if you select the same population twice, the individual is the same, and so the alleles will not be different. So $\text{Het}_{\text{fixing}}$ is $(m-1)/m$ times the probability of getting different individuals if we select them at random from *different* populations. But this is just $\frac{(m-1)}{m}h$. So

$$\text{Het}_{\text{pooling}} = \frac{1}{m}\text{Het}_{\text{averaging}} + \text{Het}_{\text{fixing}}$$

Rearranging gives

$$\text{Het}_{\text{fixing}} = \text{Het}_{\text{pooling}} - \frac{1}{m}\text{Het}_{\text{averaging}}$$
