# Supplementary material for: Measuring genetic diversity across populations
Source: PLoS Comput Biol. 2024 Dec 4;20(12):e1012651. doi: 10.1371/journal.pcbi.1012651 (PMC11649088; doi:10.1371/journal.pcbi.1012651)
Supplement: S1 Fig — Each subplot presents the correlation of two diversity functions measured on sets of populations with size 3. Each blue dot is a set of 3 populations. The x and y axes are all combinations of the population diversity measures based on Het and SSD. (PDF) [file pcbi.1012651.s006.pdf]

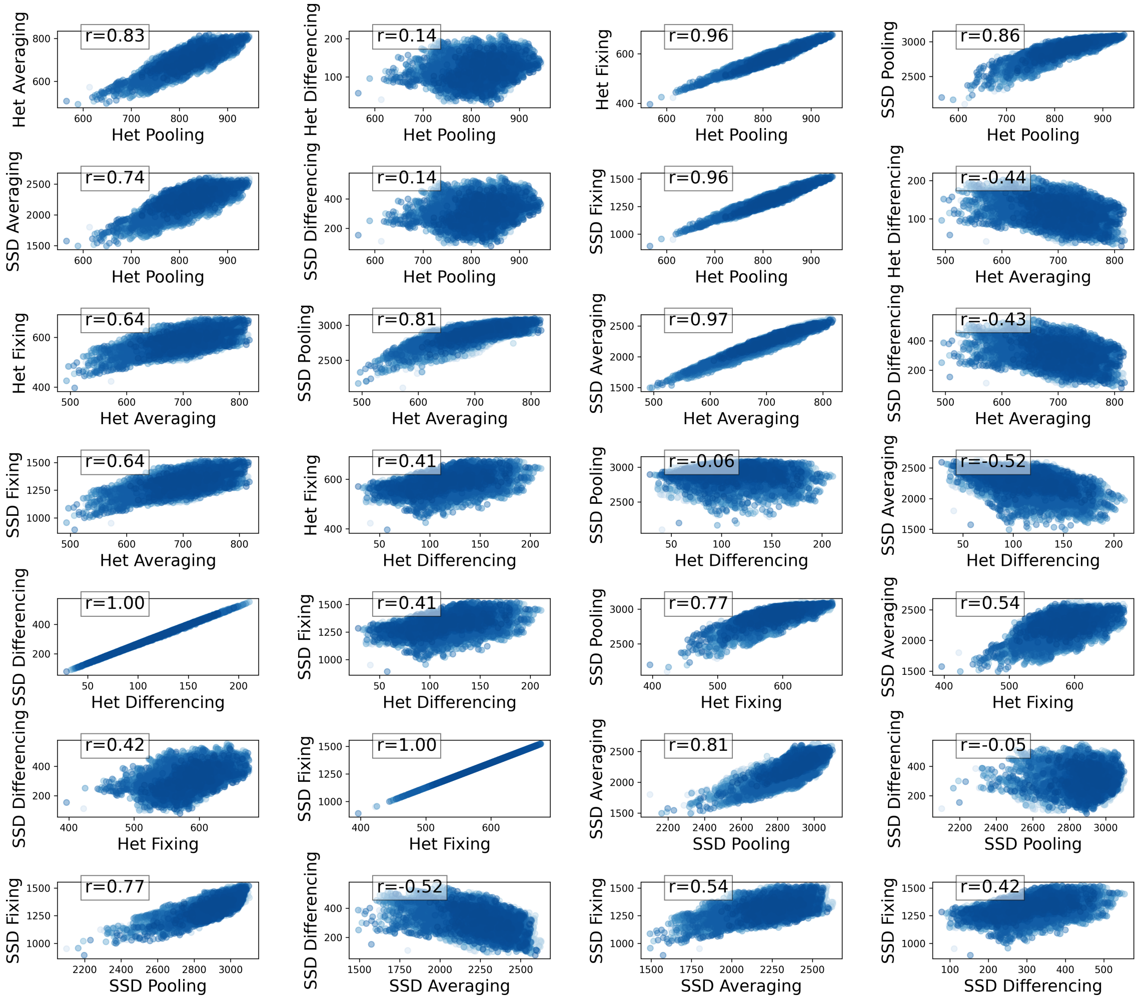

S1 Fig. Correlation of diversity metrics based on SSD and Het in Atlantic salmon. Each subplot presents the correlation of two diversity functions measured on sets of populations with size 3. Each blue dot is a set of 3 populations. The x and y axes are all combinations of the population diversity measures based on Het and SSD.
