# Supplementary material for: Measuring genetic diversity across populations
Source: PLoS Comput Biol. 2024 Dec 4;20(12):e1012651. doi: 10.1371/journal.pcbi.1012651 (PMC11649088; doi:10.1371/journal.pcbi.1012651)
Supplement: S2 Fig — Each subplot presents the correlation of two diversity functions measured on sets of populations with size 3. Each orange dot is a set of 3 populations. The x and y axes are all combinations of the population diversity measures based on Het and SSD. (PDF) [file pcbi.1012651.s007.pdf]

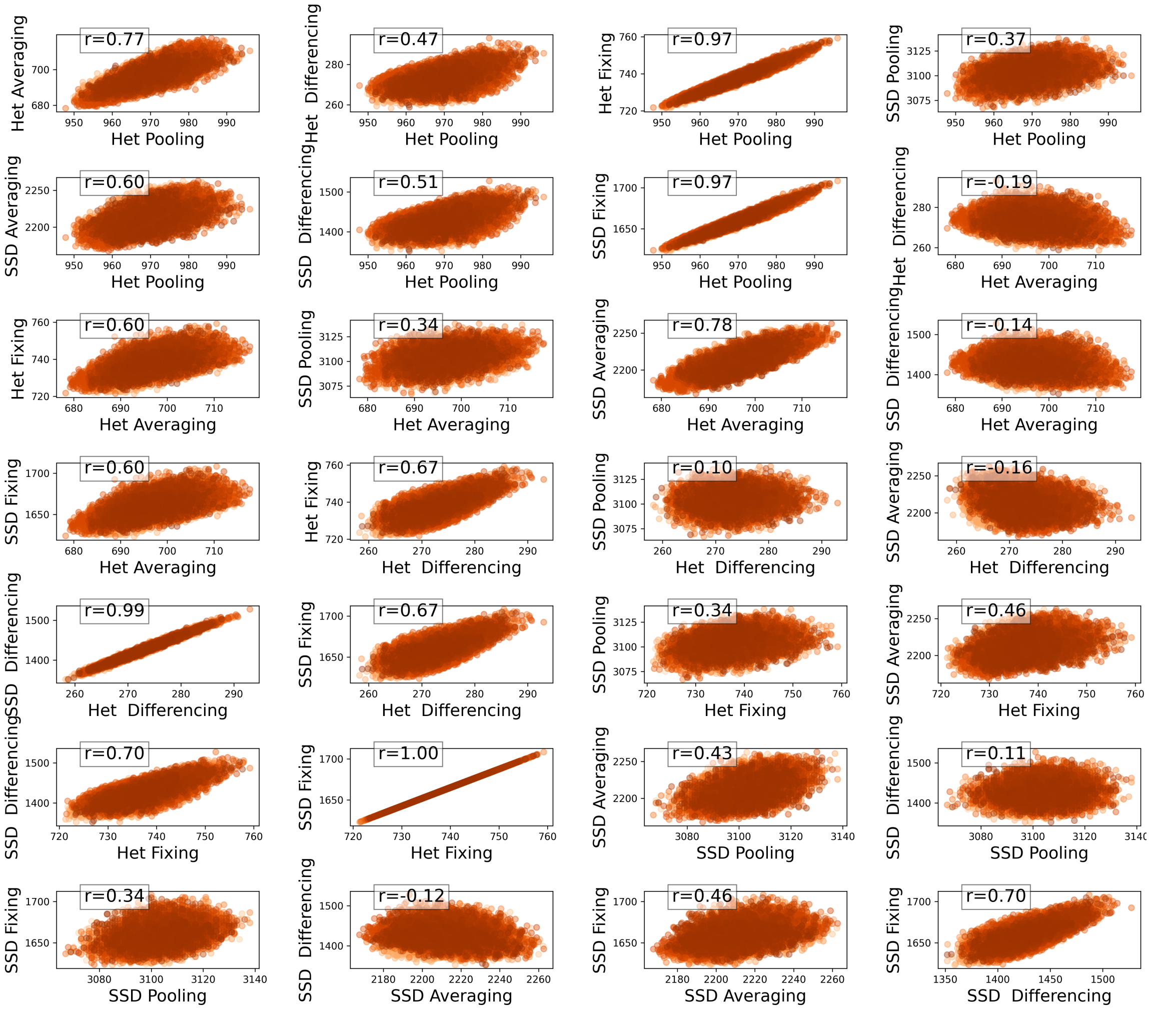

S2 Fig. Correlation of diversity metrics based on Het and SSD on a randomly permuted version of the Atlantic salmon data. Each subplot presents the correlation of two diversity functions measured on sets of populations with size 3. Each orange dot is a set of 3 populations. The x and y axes are all combinations of the population diversity measures based on Het and SSD.
