# Supplementary material for: Measuring genetic diversity across populations
Source: PLoS Comput Biol. 2024 Dec 4;20(12):e1012651. doi: 10.1371/journal.pcbi.1012651 (PMC11649088; doi:10.1371/journal.pcbi.1012651)
Supplement: S4 Fig — Each subplot shows the distribution of the standard errors of the correlations between all pairs of diversity measures in Table 2, which are computed for 1000 randomly selected subsets of size k. The distribution in red and purple corresponds to two random samples of size 1000 with different seeds. (PDF) [file pcbi.1012651.s009.pdf]

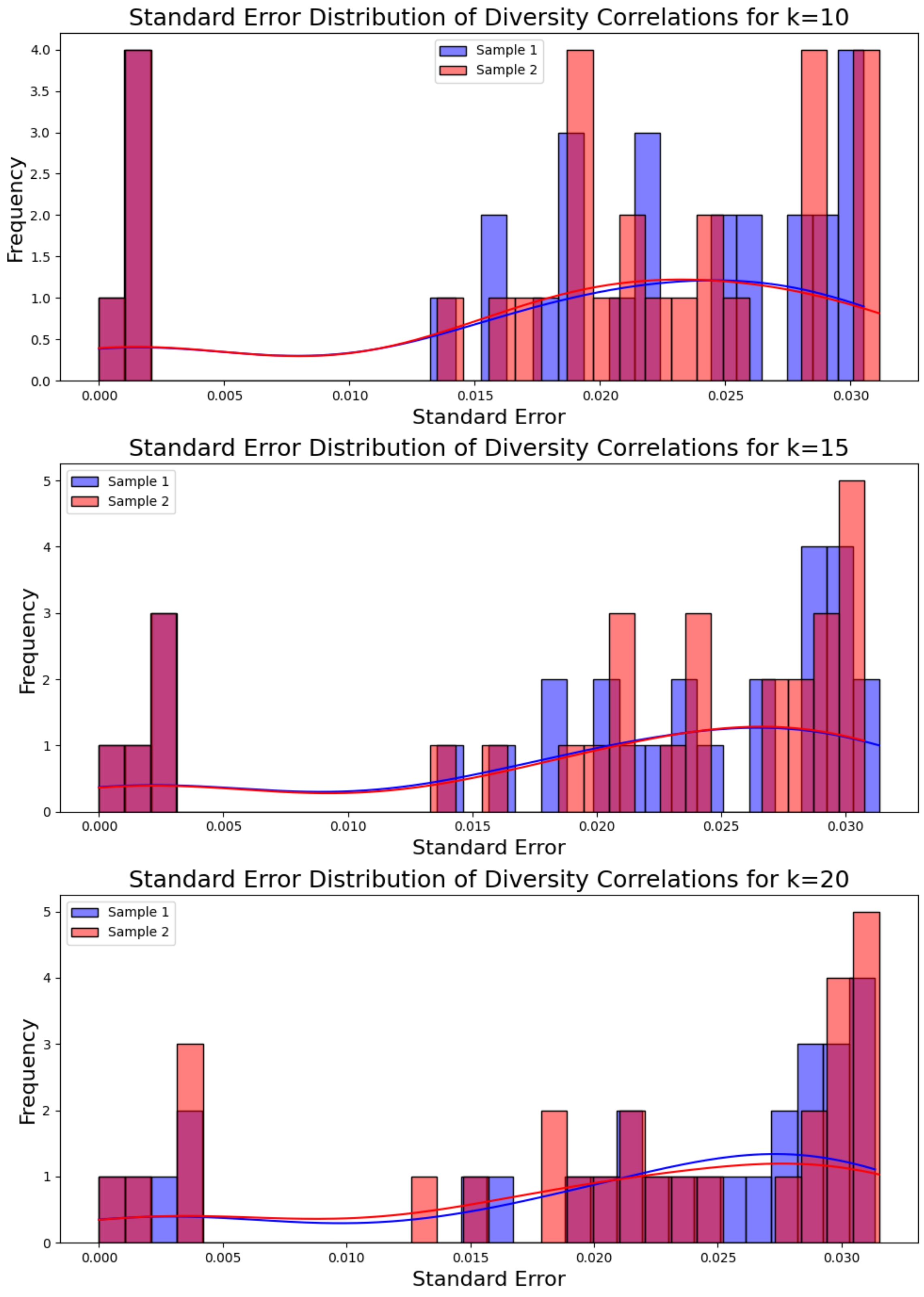

S4 Fig. Standard error of the correlations of diversity metrics for $k = 10, 15$ and 20. Each subplot shows the distribution of the standard errors of the correlations between all pairs of diversity measures in Table 2, which are computed for 1000 randomly selected subsets of size k. The distribution in red and purple corresponds to two random samples of size 1000 with different seeds.
