# Supplementary figures and images for: Measuring genetic diversity across populations
Source: PLoS Comput Biol. 2024 Dec 4;20(12):e1012651. doi: 10.1371/journal.pcbi.1012651 (PMC11649088; doi:10.1371/journal.pcbi.1012651)

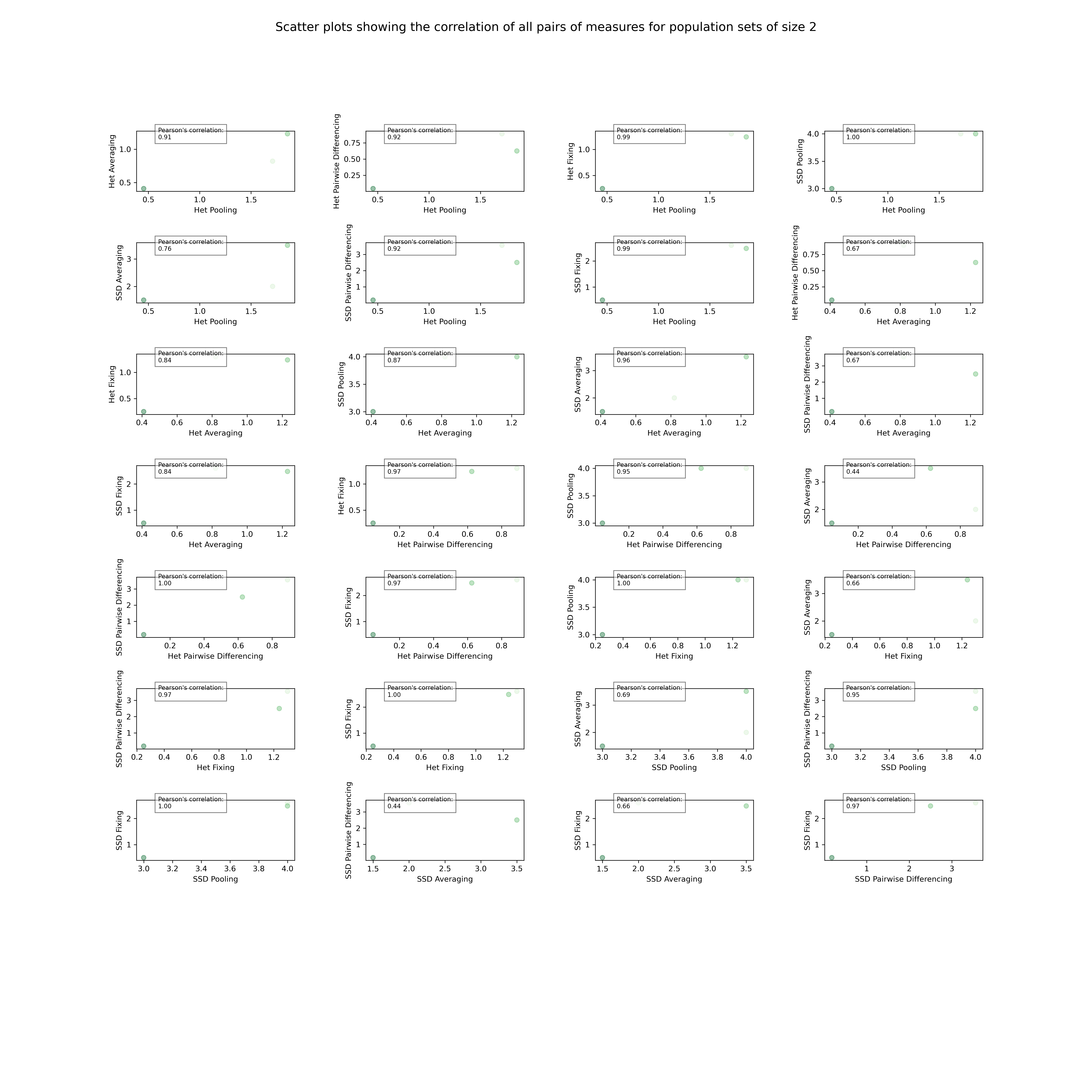

Supplement: S1 Codes — This file contains Python scripts, available in this repository, https://github.com/nabhari/population-diversity-tool, used for calculating population-based diversity measures, including Het-based and SSD-based metrics, as well as performing correlation analyses between them. The code also includes modules for brut-force search and plotting correlation results. (ZIP) [file pcbi.1012651.s010.zip › Codes/test_example_scatter.png]
